# Effects of Concomitant Benzodiazepines and Antidepressants Long-Term Use on Social Decision-Making: Results From the Ultimatum Game

**DOI:** 10.3389/fpsyg.2022.915265

**Published:** 2022-06-22

**Authors:** Carina Fernandes, Helena Garcez, Senanur Balaban, Fernando Barbosa, Mariana R. Pereira, Celeste Silveira, João Marques-Teixeira, Ana R. Gonçalves

**Affiliations:** ^1^Laboratory of Neuropsychophysiology, Faculty of Psychology and Education Sciences, University of Porto, Porto, Portugal; ^2^Department of Psychology, Istanbul Sabahattin Zaim University, Istanbul, Turkey; ^3^São João University Hospital Center, Porto, Portugal

**Keywords:** benzodiazepines, antidepressants, decision-making, unfairness, social cognition

## Abstract

Benzodiazepines and antidepressants have been shown to change responses to unfairness; however, the effects of their combined use on unfairness evaluation are unknown. This study examines the effects of concomitant benzodiazepines and antidepressants long-term use on the evaluation of fair and unfair offers. To analyze behavioral changes on responses to unfairness, we compared the performance of medicated participants and healthy controls in the Ultimatum Game (UG), both in the proposer and in the respondent role. The results showed that long-term psychotropic users had the worse economic strategy by accepting less offers than control subjects. However, in the proposer role, the unfair offers made by participants were similar between groups. The present results suggest that long-term use of psychotropic medication, specifically the combination of benzodiazepines and antidepressants, may increase the sensitivity to unfairness, resulting in higher rejection rates in conditions where this strategy is the most disadvantageous.

## Introduction

Decision-making is a crucial process in daily life and has attracted a lot of attention in research addressing how social context influences decisions and behavior. Within this scope, it has become evident that there is a tendency to punish transgressors of social norms, such as fairness or reciprocity, even if it has a personal cost (Fehr and Gächter, 2002). The act of punishing those who violate the perceived norms of the group appears to have been favored during evolution (Fehr and Gächter, 2000).

The Ultimatum Game (UG; Güth et al., 1982) is one of the most studied tasks in social decision-making, contributing to increase knowledge on the nature of human fairness (Henrich et al., 2010; Corradi-Dell’Acqua et al., 2013; Artinger et al., 2014). In the UG, one player (the proposer) suggests a way to divide a monetary sum with another player (the responder). If the responder accepts the offer, the stake is divided as suggested. If the responder rejects the offer, neither player gets anything. The payoff-maximizing strategy for the responder is to accept any amount offered, and for the proposer is to offer the smallest possible amount (Vieira et al., 2014). However, proposers are likely to offer an amount close to even split (around 40% of the total), and responders reject offers below 20% roughly half of the time (Nowak et al., 2000). Although the response can be influenced by several factors, such as genetic traits, cultural variation, and influences from social interactions (e.g., social hierarchy, reputation building, and avoidance of social rejection), the tendency to punish norm transgressors appears to prevail despite these factors (Henrich et al., 2005; Wallace et al., 2007; Gospic et al., 2011).

A study by Crockett et al. (2010) found that a single dose of antidepressants may increase acceptance rates to unfair offers during the UG. Similarly, previous studies showed that benzodiazepine treatment (consisting of a single administration of oxazepam 20 mg) decreased the rejection rate to unfair proposals even though the perception of unfairness was not affected. Moreover, a decrease in amygdala activity was found in response to unfair proposals in benzodiazepine users, while the rejection rate to unfair offers was associated to an increased amygdala activity in controls (Gospic et al., 2011). These results suggest that the limbic system seems to be involved in the act of prompt rejection, a role previously thought to be exclusive of the cortical system (Sanfey et al., 2003; Gospic et al., 2011).

It is a common practice to prescribe benzodiazepines and antidepressants simultaneously in initial depression treatment, but previous research showed that the simultaneous use extends beyond the temporary measure and becomes long term (van Dijk et al., 2002).

Taken together, the results of these studies (Crockett et al., 2010; Gospic et al., 2011) suggest that the combined use of benzodiazepines and antidepressants may have a cumulative effect in decreasing the sensitivity to unfairness, which may result in disadvantageous decisions in real-life social contexts. In such contexts, the punishment of the others’ unfairness may be needed to increase fairness in social interactions. However, as far as we know, no studies have previously investigated the effect of the concomitant use of benzodiazepines and antidepressants in social decision-making. Furthermore, studies assessing the effects of long-term use of such substances on social decision-making are also lacking. The present study aims to fill this gap in the literature, shedding light on how long-term use of the psychotropic medication can affect social decision making. To this purpose, we compared the performance of medicated participants and healthy controls during the Ultimatum Game (UG), both in the proposer role and in the respondent role.

The research on this field is a matter of paramount importance considering that benzodiazepines are the most prescribed drugs in the world (Ashton, 2005) despite their unwanted effects (e.g., psychomotor and cognitive impairments; see Lader, 1999). This class of drugs acts at the limbic system, including at the thalamic and hypothalamic levels of the central nervous system (Barker et al., 2004) through the modulation of GABA actions (Argyropoulos and Nutt, 1999). Antidepressants were reported to increase the activation of dorsolateral, dorsomedial, and ventrolateral prefrontal cortices and to decrease the activation of the amygdala, hippocampus, parahippocampal region, ventral anterior cingulate cortex, orbitofrontal cortex, and insula (Delaveau et al., 2011). Bearing in mind that some of these structures are involved in immediate punishment of unfair behavior (Gospic et al., 2011), we may expect lower rejection rates of unfair offers from long-term concomitant benzodiazepines and antidepressants users. We also expect lower offers from the group taking medication in the proposer role. Furthermore, neurocognitive measures were collected to explore whether the differences in acceptance rates between groups are associated with cognitive performance.

## Materials and Methods

### Participants

A total of 60 participants were recruited from the community and local University to two groups: a long-term (for at least 1 year) concomitant benzodiazepines and antidepressants users (experimental group) and a control group, matched on age and years of formal education. We excluded participants with scores inferior to 22 (cutoff for mild cognitive impairment; Freitas et al., 2014) in the Montreal Cognitive Assessment (MoCA; Nasreddine et al., 2005; *n* = 1), as well as participants reporting uncorrected visual impairments (*n* = 1), history of brain injury and neurological diagnosis (*n* = 2). Participants who reported use of psychotropic medication besides benzodiazepines and antidepressants (*n* = 4), as well as participants with major psychiatric diagnosis aside from anxiety and depression were also excluded from the experimental group. From the control group, we exclude participants that reported use of any psychotropic medication (*n* = 13) and psychiatric diagnosis (*n* = 4). Additionally, nine participants dropped out the study at the end of the neuropsychological assessment. Thus, the final sample was composed of 13 experimental subjects (all female; *M_age_* = 44.1, *SD* = 10.0; *M_years of education_* = 15.9, *SD* = 2.4) and 13 control subjects (12 female; *M_age_* = 46.5, *SD* = 10.9; *M_years of education_* = 16.9, *SD* = 4.9).

### Instruments and Tasks

#### Self-Report Measures

Anxiety and depression traits were evaluated by the Hospital Anxiety and Depression Scale (HADS; Snaith and Zigmond, 1994; Portuguese version by Pais-Ribeiro et al., 2007), and the Brief Symptom Inventory (BSI; Derogatis, 1993; Portuguese version by Canavarro, 1999) was administered to measure psychopathological symptomatology.

#### Neuropsychological Measures

Executive functioning was assessed through the Trail Making Test (TMT; Armitage, 1946; normative data by Cavaco et al., 2013a), and the INECO Frontal Screening (IFS; Torralva et al., 2009; Portuguese version by Moreira et al., 2014). Visuospatial short-term memory was assessed by the Corsi Block-Tapping Task (CBTT; Wechsler, 2008). Phonemic and semantic fluency tests were used to assess non-motor processing speed, language production, and executive functions (Strauss et al., 2006; Portuguese versions by Cavaco et al., 2013b).

#### Experimental Task

**Figure 1:**
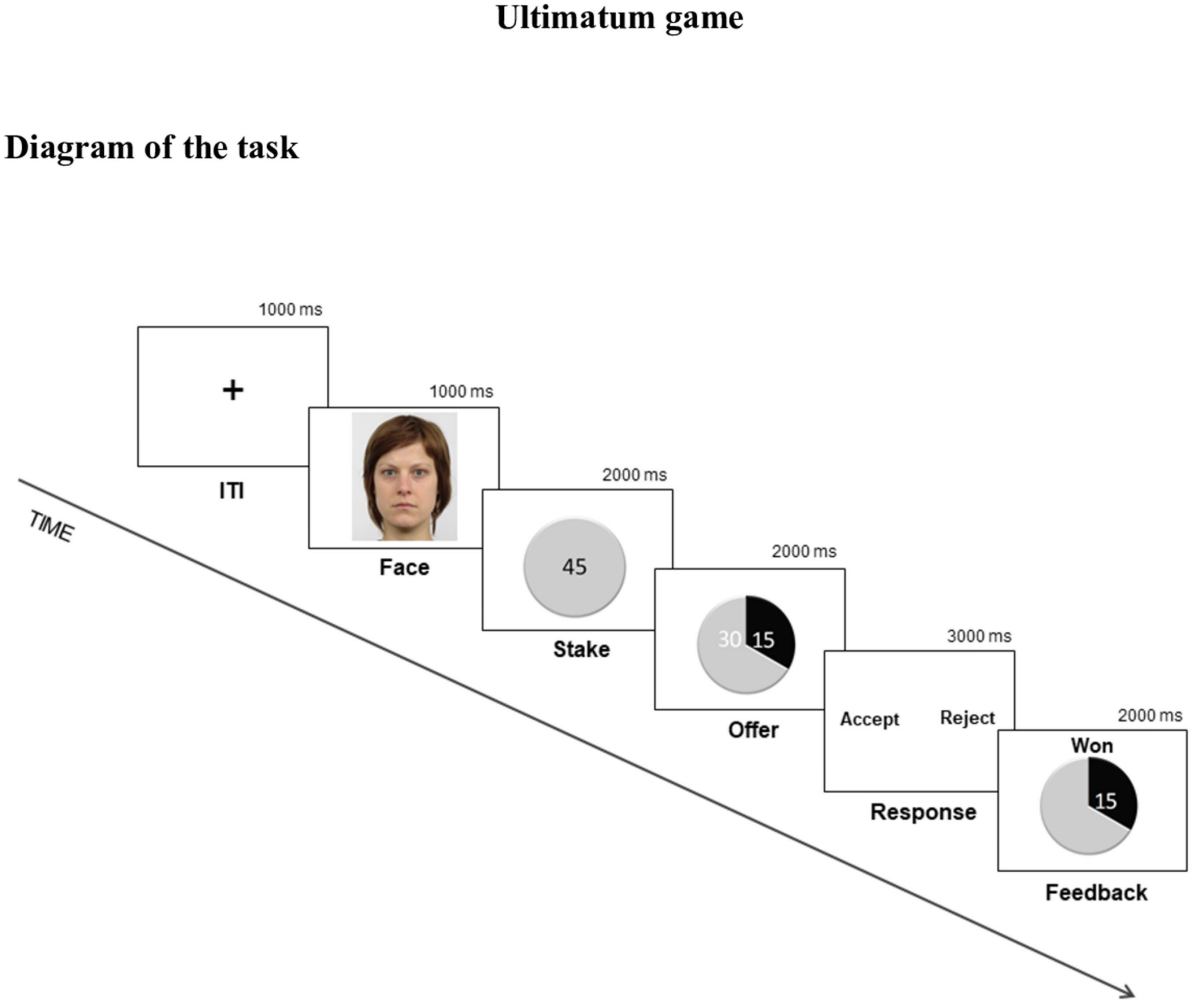
Schematic representation of one round of the Ultimatum Game (UG; dark-gray portion = respondent; light-gray portion = proposer); ITI, intertrial interval. Figure reproduced with permission from the Radboud Faces Database (RaFD).

In series of one-shot UG, participants played as respondents. Participants viewed offers and photos, told to be from previous participants, and were asked to accept or reject each offer. Photos of other players were selected from the Radboud Faces Database (Langner et al., 2010) and displayed Caucasians with direct eye contact, closed mouth, and neutral facial expression. The stake was displayed after the proposer’s photo and the offer appeared immediately after. Participants responded during a response slide, using a response box to either accept or reject the offer. At the end of each round, a feedback slide was displayed (Figure 1).

Participants played 84 rounds composed of 42 unfair and 42 fair offers. The offered amounts were 5 or 15, and the stake size varied, following previous paradigms (Crockett et al., 2008; Vieira et al., 2014; Table 1). Each stake was repeated 14 times. E-Prime 2.0 (2011, Psychology Software Tools, Inc., Sharpsburg, PA, United States) was used to create the task and collect responses.

**Table 1:** Ultimatum Game offers.

Amount offered	Stake size	
	Unfair	Fair
5	15	10
	20	11
	25	12.5
15	45	30
	60	33
	75	37.5

### Procedures

The current study was part of a larger research project approved by the local Ethics Committee. Participants gave their written, informed consent and received 20€ (gift card) as compensation for their time and travel expends. All participants were tested individually in two experimental sessions to avoid fatigue effects. In the first session, it was conducted a semi-structured interview, followed by the administration of the MoCA to assess inclusion criteria. The remaining neuropsychological tests and self-report measures were then administered in a random order between participants. Participants who fulfilled the inclusion criteria were invited to participate on a second session, in which the experimental task was performed. Participants were informed that the offers were made by previous players and that their offers would be presented to the succeeding participants (Crockett et al., 2008; Vieira et al., 2014). As respondents, participants viewed the rounds on a computer screen from a distance of 115 cm. After four practice trials, participants completed 84 experimental trials presented randomly. Afterward, participants made their offers (proposer role) and rated the fairness of each offer received (respondent role), using a Likert scale (1 = very unfair; 7 = very fair).

### Statistical Analysis

The neuropsychological and self-report results were compared between groups with Student’s *t* tests. Whenever necessary, equivalent non-parametric tests were performed. The acceptance rates were calculated by participant and condition (fair and unfair offers) for the respondent role. The effects of offer type and group on the acceptance rates and reaction times were analyzed in separate mixed ANOVAs. The *type of offer* (fair, unfair) was used as within-subjects factor, and *group* (experimental, control) was used as between-subjects factor.

To investigate the effects of concomitant benzodiazepines and antidepressants long-term use on *perceived fairness*, we compared fairness ratings between groups through a mixed factors ANOVA, with *type of offer* as within-subjects factor and group as between-subjects factor. To analyze the effects of concomitant benzodiazepines and antidepressants long-term use on the proposer role, the amount offered (%) by experimental and control subjects was compared with Student’s *t* test.

The correlations between neurocognitive and behavioral results were explored through Spearman Correlations (Rousselet and Pernet, 2012).

The threshold for statistical significance was set at *α* = 0.05, and the *p* values reported for *t*-tests are from one-tailed tests. Statistical analysis was performed using SPSS 24 (IBM Corp., Armonk, NY, United States). Violations of sphericity in ANOVA were corrected *via* the Greenhouse–Geisser method.

## Results

### Neuropsychological Results

Neuropsychological data analysis revealed no differences between groups in IFS, CBTT, TMT, PF, nor SF (all *p* > 0.313). However, significant group differences were observed on depression, *t*(24) = −3.735, *p* < 0.002, anxiety, *t*(24) = −3.156, *p* = 0.004, and psychopathological symptomatology, *t*(24) = −4.450, *p* < 0.001. Experimental subjects had higher scores in these self-report measures (see Table 2).

**Table 2:** Mean (and SD) values of sociodemographic data, neuropsychological tests, and UG task of the participants.

	Control (*n* = 13)	Experimental (*n* = 13)
**Neuropsychological data**		
MoCA	27.3 (1.7)	25.2 (2.2)
IFS	23.3 (2.3)	23.7 (3.5)
CBTT	16.0 (3.9)	15.1 (3.5)
TMT	52.1 (28.9)	50.9 (25.5)
Phonemic fluency	42.2 (15.3)	36.5 (12.4)
Semantic fluency	20.1 (6.3)	20.2 (6.4)
HADS depression	3.3 (2.1)	9.2 (5.2)
HADS anxiety	6.2 (3.1)	11.0 (4.5)
BSI	29.6 (15.4)	79.2 (37.1)
**Ultimatum Game**		
Acceptance rates of fair offers	90.5 (11.8)	69.4 (21.9)
Acceptance rates of unfair offers	25.8 (26.4)	15.8 (16.5)
Reaction times of fair offers	659 (161)	633 (236)
Reaction times of unfair offers	693 (151)	657 (295)
Fairness ratings of fair offers	5.59 (0.77)	5.55 (0.82)
Fairness ratings of unfair offers	2.87 (1.02)	2.56 (1.12)
Amount offered	44.5 (3.65)	46.6 (5.56)

MoCA, Montreal Cognitive Assessment; IFS, Institute of Cognitive Neurology Frontal Screening; CBTT, Corsi Block-Tapping Task; TMT, Trail Making Test; HADS, Hospital Anxiety and Depression Scale; and BSI, Brief Symptom Inventory.

### Behavioral Results

Descriptive statistics for the acceptance rates, reaction times, fairness ratings, and amounts offered are shown in Table 1. The results showed a main effect of *group* for acceptance rates, *F*(1, 24) = 274.4, *p* = 0.017, η^2^_p_ = 0.215, *ε* = 0.690, revealing that control subjects accepted offers more often than experimental subjects. Additionally, a main effect of *type of offer*, *F*(1, 24) = 144.4, *p* < 0.001, η^2^_p_ = 0.858, *ε* = 1.00, showed that fair offers were accepted more often than unfair offers. The interaction type of offer*group was non-significant, *F*(1, 24) = 1.24, *p* = 0.275. There was a negative correlation between the IFS score and the acceptance of unfair offers for the control group, *r(*11) = −0.58, *p* = 0.037. The remaining correlations between the acceptance rates and the neurocognitive results were non-significant (all *p* > 0.171).

Regarding reaction times, no main effect of *group*, *F*(1, 24) = 0.15, *p* = 0.705, or *type of offer*, *F*(1, 24) = 0.86, *p* = 0.362, were observed. The interaction offer*group was also non-significant, *F*(1, 24) = 0.02, *p* = 0.875.

The analysis of the perceived fairness ratings revealed no main effects of *group*, *F*(1, 24) = 0.33, *p* = 0.570. However, a main effect of *type of offer* emerged, *F*(1, 24) = 172.5, *p* < 0.001, η^2^_p_ = 0.878, *ε* = 1.00, with fair offers being rated as more fair than unfair offers. The interaction offer*group was non-significant, *F*(1, 24) = 0.39, *p* = 0.539.

In the proposer role, the amount offered was similar between groups, *t*(24) = −1.155, *p* = 0.260.

## Discussion

In the present study, long-term concomitant benzodiazepines and antidepressants users and control subjects performed the UG as proposers and respondents. With this approach, we aimed to examine the effects of the combined used of such medication, for a long period, on the evaluation of fair and unfair offers.

Our results showed that control subjects followed a more advantageous economic strategy accepting more offers than the experimental group. However, the lower rejection rates of unfair offers from the medicated group, hypothesized from the results of previous studies (Crockett et al., 2010; Gospic et al., 2011), were not confirmed. On the contrary, participants from the experimental group rejected more fair and unfair offers, despite the similar perception of unfairness between groups. This result is opposite to the one reported by a previous study that examined the involvement of the amygdala in the rejection of unfair offers, through a pharmacological intervention (Gospic et al., 2011). In this study, a single administration of oxazepam 20 mg decreased the rejection rate of unfair offers simultaneously with a decreased amygdala activity in response to unfair proposals (Gospic et al., 2011), suggesting the involvement of the limbic system in the processing and prompt rejection of unfair offers. Moreover, a former study showed that a single dose of antidepressant decreased the rejection rate of unfair offers during the UG (Crockett et al., 2010). This result is consistent with the evidence that antidepressants decrease the activation of the amygdala and other limbic structures (Delaveau et al., 2011), while increasing the activation of dorsolateral, dorsomedial, and ventrolateral prefrontal cortices (Delaveau et al., 2011). Noteworthy, these studies assessed the effects of benzodiazepines and antidepressants on social decision-making when used alone and in a single administration, whereas our study examined the combined and long-term effects of these drugs. When these psychotropic drugs are used together, their interactions may affect the brain structures in a different way, resulting in behavioral effects distinct from the cumulative ones that we were expecting.

The current sample of concomitant benzodiazepines and antidepressants users scored higher in self-report measures of anxiety, depression, and psychopathological symptomatology. The performance in these tests could have contributed for the acceptance rates on the UG. In fact, rejection in this task has been previously linked to depression (Gradin et al., 2017). However, our results showed that performance on the UG was not associated with the self-report measures.

Bearing in mind that longer deliberation has been linked with more advantageous economic decisions (Rand et al., 2012); we analyzed reaction times to clarify if the similar acceptance rates of unfair offers could be attributed to longer reaction times in the experimental group. It could indicate higher cognitive effort to overrule negative emotional responses to unfairness, or lower impulsivity when facing the conflict of between losing money and accepting unfair offers. However, the groups had similar reaction times. Nonetheless, the acceptance rates of unfair offers in the control group were negatively correlated with the performance in a test assessing executive functioning (IFS). Previous studies examining the influence of cognitive resources on the acceptance rates for unfair offers found diverse effects (Hallsson et al., 2018). This suggests that other factors introducing variability may affect the association between cognitive resources and prosocial behavior (Krajbich et al., 2015). For example, Harris et al. (2020) suggested that lower acceptance rates for the unfair offers indicate a deliberate strategy to override the appeal of short-term financial gain to preserve social standing.

Regarding the proposer role, the two groups offered similar amounts of money, contrary to our hypothesis. Our prediction was based on the evidence that the use of benzodiazepines facilitates aggressive (Hall and Zisook, 1981) and violent behavior (Dåderman and Lidberg, 1999; Dåderman et al., 2002), suggesting inhibitory effects on empathic responses, along with the findings that antidepressant treatment reduces behavioral and neural responses to pain empathy (Rütgen et al., 2019). In view of this, we expected lower offers from the experimental group than the control group. Although empathy was not manipulated in the present task, it has been previously suggested to play a crucial role in social decision-making tasks (Beadle et al., 2013).

It should be noted that there are several factors related to the combined use of psychotropic medication that may influence the evaluation of fair and unfair offers, such as the type of antidepressants (selective serotonin reuptake inhibitors—SSRIs, serotonin-noradrenaline reuptake inhibitors, tricyclic antidepressants—TCAs, noradrenergic and specific serotonergic antidepressants, monoamine oxidase inhibitors, and other antidepressants) and benzodiazepines (short-acting or long-acting), the dose of each drug, and the percentage of days of concomitant drug use. However, a larger sample would be necessary to test these variables as potential moderators of any concomitant drug use effects observed. The literature in the present field is scarce and multi-center studies addressing the influence that long-term use of benzodiazepines and antidepressants may have on social cognition are needed.

Another limitation of the study is the lack of randomized groups as found in Gospic et al. (2011) and Crockett et al. (2010), in which a placebo had been given to the control group and a drug was administered to the experimental group. This randomized design allows inferring that the effects observed were related to the treatment. In our study, the experimental group is composed of subjects using simultaneously benzodiazepines and antidepressants for at least 1 year making any deductions of the effects less clear. However, to control differences between the groups, a set of neuropsychological tests and self-report measures was administered to participants and the results from both groups were compared. Furthermore, we analyzed whether performance on the UG was associated with performance in these tests. The randomization of groups to assess long-term effects is a limitation of the studies in this field; however, it is essential to explore the effects of two widely prescribed drugs bearing the shortcomings in mind.

In sum, despite the above-mentioned shortcomings, the results suggest that the combined long-term use of benzodiazepines and antidepressants affect social decision-making as measured by the UG, with individuals using these drugs deciding less often for the best economic strategy. Nevertheless, further studies within this field are necessary to corroborate this finding. We hope, however, that the present findings impact future research on social cognition under the influence of two commonly used drugs.

## Data Availability Statement

The raw data supporting the conclusions of this article will be made available by the authors, without undue reservation.

## Ethics Statement

The studies involving human participants were reviewed and approved by Comissão de ética da Faculdade de Psicologia eCiências da Educação da Universidade do Porto. The patients/participants provided their written informed consent to participate in this study.

## Author Contributions

AG designed the study, collected and analyzed the data, and wrote the manuscript. CF designed the study. HG collected and analyzed the data. SB analyzed the data. FB, MP, CS, and JM-T assisted in designing the study. All authors contributed to the article and approved the submitted version.

## Funding

This research was supported by Norte Portugal Regional Operational Program (NORTE 2020), under the PORTUGAL 2020 Partnership Agreement, through the European Regional Development Fund (ERDF), and by national funds, through the Foundation for Science and Technology, IP (FCT), NORTE-01-0145-FEDER-028038—PTDC/PSI-ESP/28038/2017 and NORTE-01-0145-FEDER-029435—PTDC/PSI-GER/29435/2017.

## Conflict of Interest

The authors declare that the research was conducted in the absence of any commercial or financial relationships that could be construed as a potential conflict of interest.

## Publisher’s Note

All claims expressed in this article are solely those of the authors and do not necessarily represent those of their affiliated organizations, or those of the publisher, the editors and the reviewers. Any product that may be evaluated in this article, or claim that may be made by its manufacturer, is not guaranteed or endorsed by the publisher.
